# Clinical Soft Tissue Adaptation to Biomechanical Modulation with the Bone Protection System (BPS): A Two-Case Report in Thin-Biotype Patients

**DOI:** 10.3390/jcm15020721

**Published:** 2026-01-15

**Authors:** Anna Ewa Kuc, Jacek Kotuła, Kamil Sybilski, Grzegorz Hajduk, Joanna Lis, Beata Kawala, Michał Sarul, Magdalena Sulewska

**Affiliations:** 1 Department of Dentofacial Orthopedics and Orthodontics, Wroclaw Medical University, 50-425 Wroclaw, Poland; j_kotula@poczta.onet.pl (J.K.); joanna.lis@umw.edu.pl (J.L.); beata.kawala@umw.edu.pl (B.K.); 2 Faculty of Mechanical Engineering, Military University of Technology, 00-908 Warsaw, Poland; kamil.sybilski@wat.edu.pl; 3 Chair and Department of Oral Surgery, Medical University of Lublin, Doktora Witolda Chodźki 6 Street, 20-093 Lublin, Poland; esso53@wp.pl; 4 Department of Integrated Dentistry, Wroclaw Medical University, 50-425 Wroclaw, Poland; michal.sarul@umw.edu.pl; 5 Department of Periodontal and Oral Mucosa Diseases, Medical University of Bialystok, Waszyngtona 13, 15-269 Bialystok, Poland; magdalena.sulewska@umb.edu.pl

**Keywords:** bone protection system, orthodontic expansion, buccal plate, thin gingival phenotype, periodontal phenotype

## Abstract

**Background:** Patients with a thin gingival phenotype and a narrow buccal alveolar plate are highly susceptible to periodontal complications during orthodontic expansion. Traditional biomechanics often fail to maintain root control in thin alveolar housing. This report presents two clinical cases illustrating soft- and hard-tissue responses to a novel biomechanical approach, the Bone Protection System (BPS), designed to reduce buccal cortical overload during expansion. **Case Presentation:** Two adult patients with a thin gingival phenotype assessed by a standardized periodontal probe transparency test and narrow alveolar ridges underwent orthodontic expansion. Patient 1 was treated with the full BPS protocol in both arches. Patient 2 received BPS only in the maxilla, while the mandible was treated conventionally, creating an intra-individual control model under identical systemic conditions. Soft-tissue phenotype and cortical plate response were evaluated clinically and radiographically when applicable. **Results:** In Patient 1 clinically, the vestibular phenotype showed clear thickening and stabilization. In Patient 2, the maxillary arch treated with BPS exhibited progressive thickening of the vestibular phenotype, whereas the mandible treated conventionally presented thinning and increased translucency—features consistent with buccal compression in thin alveolar bone. No soft- or hard-tissue augmentation procedures were performed in either case. **Conclusions:** The Bone Protection System may contribute to improved periodontal safety during orthodontic expansion in thin-biotype patients by reducing buccal cortical loading and supporting adaptive soft-tissue and bone responses. Preliminary observations suggests that BPS has potential value for possibly expanding the biological limits of safe tooth movement. Further studies on larger cohorts are warranted.

## 1. Introduction

Transverse deficiencies, thin periodontal phenotype, and reduced buccal alveolar housing are recognized risk factors in orthodontic treatment, particularly during transverse expansion [1,2,3,4]. In such patients, conventional expansion mechanics may increase compressive loading on the buccal cortical plate, which has been associated with the development of alveolar bone dehiscence, fenestration, or gingival recession [2,5,6,7,8]. Because thin-biotype patients frequently present with pre-existing cortical thinning, the limits of safe transverse dental movement are substantially reduced [1,2,9].

The periodontal phenotype plays a decisive role in determining susceptibility to bone loss and soft-tissue recession. A thin phenotype is associated with diminished buccal bone thickness, reduced soft-tissue volume, and increased translucency, all of which lower the threshold for mechanical injury [7,8,10]. Clinical and CBCT-based studies confirm that thin biotypes exhibit a higher probability of developing dehiscence during alignment and expansion [1,2,8,9,11]. Because soft-tissue thickness is strongly correlated with the underlying cortical morphology, any treatment that improves root positioning and reduces buccal overload may simultaneously enhance soft-tissue stability [1,2,3,4,5,6,7,8,9,10,11,12,13].

The periodontal ligament (PDL) responds differently to compressive and tensile forces, wherein excessive compression impairs blood flow, induces hyalinization, and boosts osteoclastic activity, while tensile strain promotes osteoblastic differentiation via mechanotransduction pathways such as integrin signaling, cytoskeletal deformation, and upregulation of RUNX2, ALP, and osteocalcin [14,15,16,17]. From a biologic standpoint, tension-supported tooth movement results in more stable alveolar adaptation, whereas compression-dominant movement predisposes to thinning or loss of the buccal plate [14,15,16,17,18].

Despite advances in skeletal anchorage and corticotomy-assisted approaches, no existing biomechanical system specifically aims to redirect force vectors during expansion to reduce buccal compression and enhance tension-dominant PDL loading. This gap is clinically significant: in thin-biotype patients, the alveolar envelope often becomes the primary limiting factor determining how far roots can safely be moved [19,20,21,22,23].

The Bone Protection System (BPS) was conceptualized as a biomechanical adjunct intended to address limitations in orthodontic expansion of thin-biotype patients by aiming to stabilize the vestibular segment during alignment and expansion, with the rationale of reducing unwanted tipping forces and promoting more physiologic displacement patterns, such as reduced uncontrolled buccal tipping on thin round wires and an improved axial trajectory. Here, ‘physiologic displacement patterns’ are used conceptually to describe tooth movement in which periodontal stresses are less concentrated cervically and are more evenly distributed along the root, potentially shifting the local balance away from compression peaks toward tension-supported remodeling. Preliminary case observations suggest an association with improved soft-tissue stability and the absence of clinically evident cortical breakdown in patients treated with BPS.

This report illustrates the application of the Bone Protection System (BPS) in two adult patients to explore its potential for adaptive soft- and hard-tissue responses during orthodontic expansion. The report is hypothesis-generating and aims to describe a clinical soft-tissue phenotype response observed during orthodontic expansion with a biomechanical adjunct (BPS), rather than to evaluate the efficacy of a therapeutic system. The aim of this case series is to present the clinical and radiographic outcomes of BPS-assisted expansion in two thin-phenotype patients, including intra-arch phenotype comparison in Patient 2.

## 2. Case Presentation

### 2.1. Patient 1

**Figure 1:**
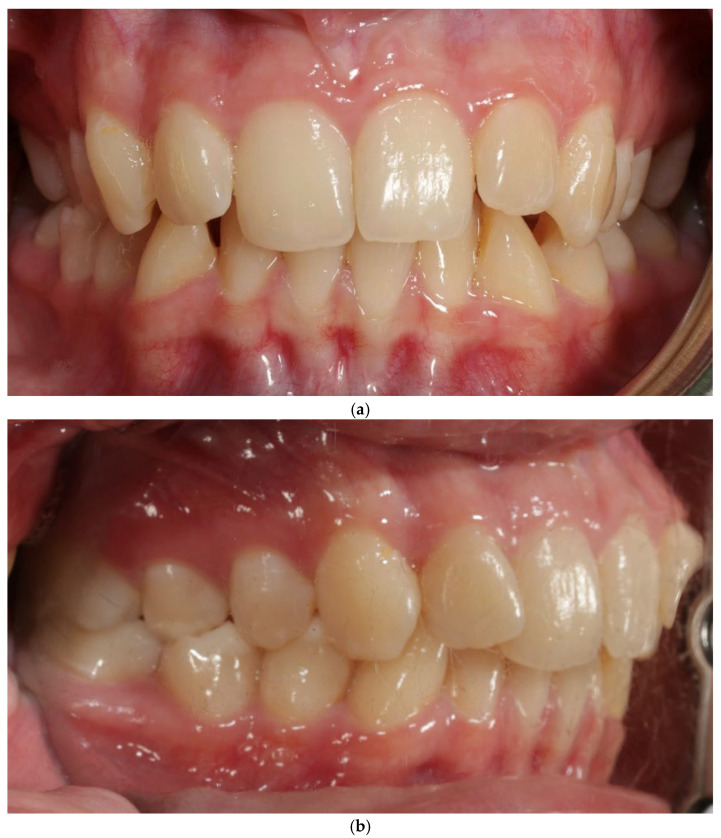

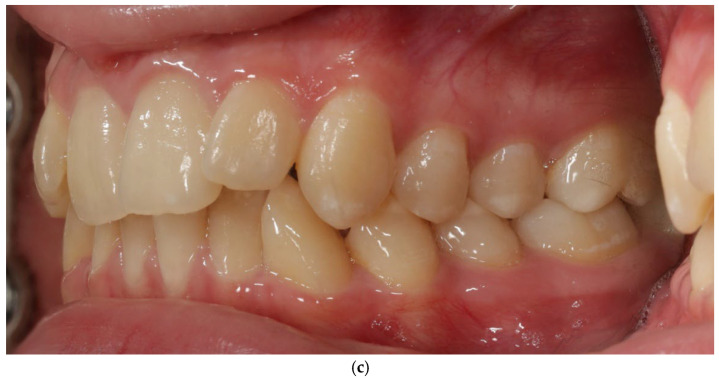
Patient 1—before treatment (**a**–**c**).

A 27-year-old female patient presented with a thin periodontal phenotype based on probe-transparency test, mild crowding in both arches, and narrow alveolar anatomy confirmed clinically and radiographically—Figure 1a–c. Thin phenotypes are known to present a significantly increased risk of buccal bone loss during expansion, especially when the cortical plate is critically thin at baseline [1,24,25,26].

Orthodontic treatment aimed to correct transverse deficiency and crowding while minimizing buccal cortical stress. A self-ligating fixed appliance was used for alignment with 4 mm expansion in the interpremolar region, and the Bone Protection System (BPS) was applied in both arches as a stabilizing adjunct for the vestibular segment, with the intent of limiting non-physiologic buccal tipping forces (Figure 1). The BPS was applied based on its proposed biomechanical rationale of modifying force distribution during expansion, rather than to demonstrate a specific biological effect in this study [27,28,29].

**Figure 2:**
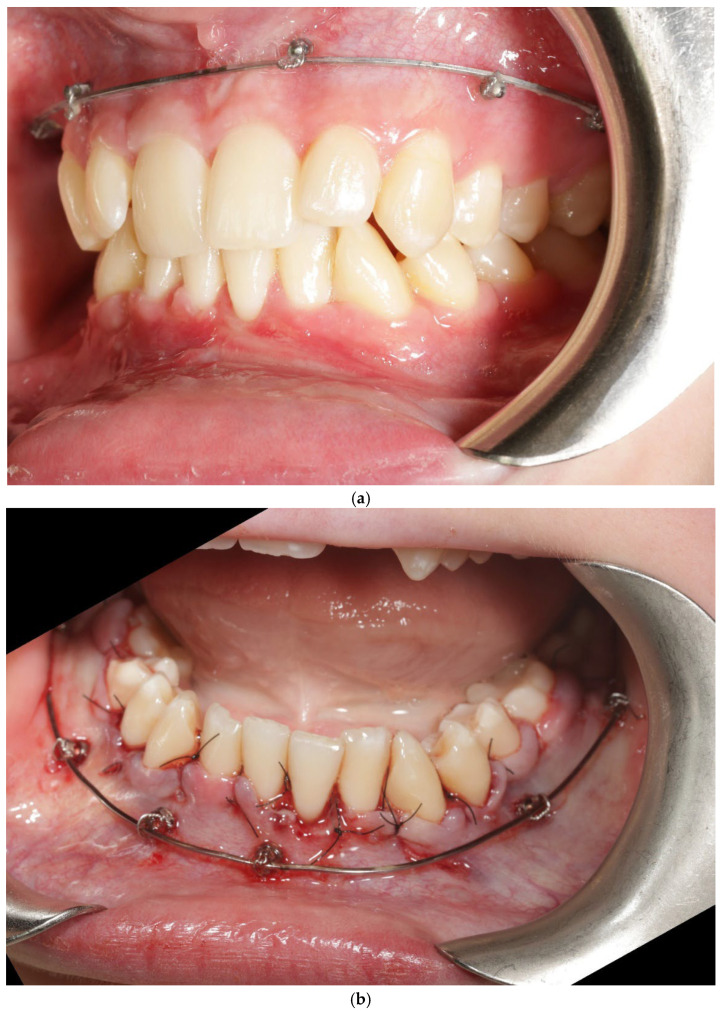
Patient 1 with Bone Protection System in both arches (**a**,**b**).

**Bone Protection System (BPS).** The Bone Protection System (BPS) is a chairside orthodontic adjunct designed to stabilize the vestibular segment during alignment and expansion (Figure 2a,b). Its purpose is to reduce non-physiologic buccal tipping moments and to guide a more axial trajectory of root movement. BPS does not apply active force; instead, it modifies the moment–force environment generated by the archwire–bracket complex by limiting uncontrolled buccal displacement of the crowns. The device is attached passively to the vestibular brackets and remains in place throughout the expansion/alignment phase. Full technical construction details are protected by a filed patent application; therefore, only clinical handling and rationale are described. The system was applied in the first months of orthodontic treatment during leveling and expansion in conjunction with the regional acceleratory phenomenon (RAP). A modified corticotomy was performed, followed by placement of orthodontic mini-implants at seven sites in the buccal segment of the maxilla. An expanded orthodontic stainless-steel archwire was engaged with the mini-implants to deliver the planned expansion mechanics and force vectors. Descriptions of biological responses to these mechanics are beyond the scope of this case report and are addressed in the Discussion. Detailed FEM analyses are reported in a separate bioengineering manuscript under review; therefore no FEM datasets or numerical outputs are reproduced in this case report.

Orthodontic treatment was performed using a self-ligating fixed appliance (0.022″ MBT prescription). Archwire sequence included 0.014″ NiTi, 0.018″ NiTi, 0.014 × 0.025″ NiTi, and 0.019 × 0.025″ TMA. Intercanine expansion was approximately 2 mm and intermolar expansion 4 mm. No extractions or IPR were performed.

#### Clinical Soft-Tissue Findings

Clinically, the vestibular phenotype appeared improved, with reduced translucency, increased convexity, and greater soft-tissue resistance to palpation; no recession or inflammation was observed.

Soft-tissue improvement occurred spontaneously, without any mucogingival surgery. This pattern is consistent with the known relationship between buccal bone form and soft-tissue phenotype [1,24,30,31,32,33].

### 2.2. Patient 2

**Figure 3:**
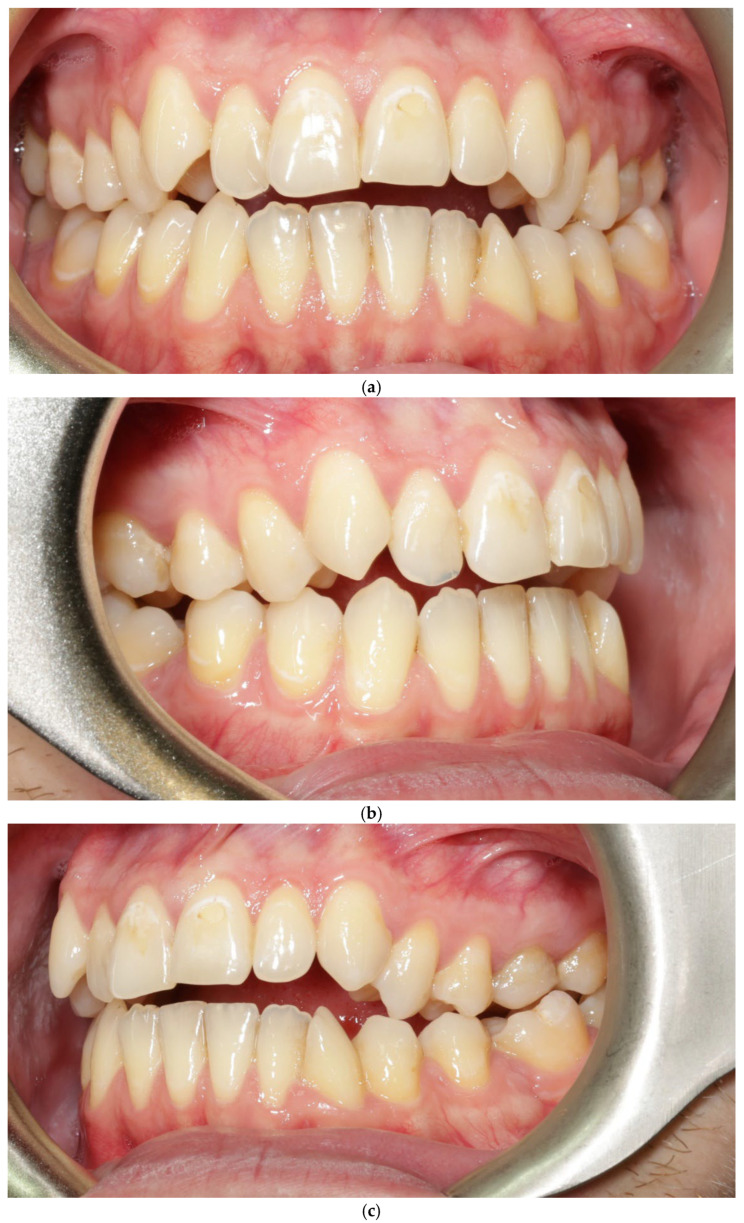
Patient 2 before treatment (**a**–**c**).

A 22-year-old female patient presented with: anterior open bite, maxillary constriction, crowding and a thin periodontal phenotype—Figure 3a–c.

In this patient, BPS was applied only in the maxillary arch with cross bite, while the mandible underwent conventional mechanics. This created an intra-patient cross-arch control, allowing direct comparison of the biological response under identical systemic conditions. Orthodontic treatment was performed using a self-ligating fixed appliance (0.022″ MBT prescription). Archwire sequence included 0.014″ NiTi, 0.018″ NiTi, 0.014 × 0.025″ NiTi, 0.018 × 0.025″ NiTi (all PITZ form) and 0.019 × 0.025″ TMA. Intercanine expansion was approximately 3 mm and intermolar expansion 5 mm. No extractions or interproximal reduction (IPR) were performed.

#### 2.2.1. Clinical Phenotype Comparison

The maxillary BPS arch demonstrated a thicker soft-tissue phenotype, decreased translucency, more pronounced vestibular convexity, and a stable gingival margin. The mandibular arch (control) demonstrated a persistent thin, translucent phenotype, flattened contour, and no reinforcement.

Findings from both patients are summarized in Table 1.

#### 2.2.2. Radiology—CBCT Timing and Limitations

A CBCT scan was obtained immediately after removal of BPS to verify cortical continuity.

However, metal scatters from brackets and archwire, and the fact that the scan captured only the early remodeling phase, made thin buccal cortices partially invisible due to lack of mineralization. As CBCT detects only mineralized tissue, quantitative assessment was not performed. A full post-treatment CBCT will be obtained after orthodontic finishing, when newly formed bone becomes radiographically detectable [34,35,36,37]. CBCT imaging was performed using a standard dental CBCT unit with a limited field of view and high-resolution settings appropriate for alveolar bone assessment. Given the presence of orthodontic appliances, metal-related artifacts were anticipated. To mitigate their impact, image interpretation was restricted to regions where the buccal cortical plate was clearly visible and not obscured by scatter, and no quantitative measurements were attempted. All CBCT findings in this report are therefore qualitative and were interpreted conservatively, within the limits of scan interpretability.

Periodontal safety:

No recession, bleeding on probing, or inflammation was observed.
No phenotype augmentation or bone grafting procedures were performed.
All changes in the maxilla occurred purely biomechanically.

## 3. Results

### 3.1. Patient 1—Clinical Outcomes

**Figure 4:**
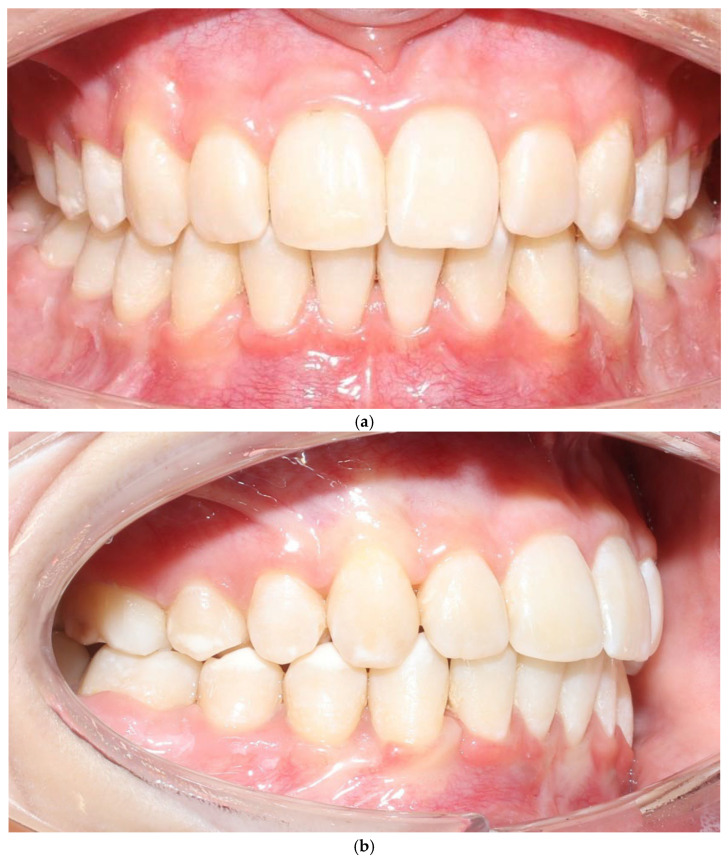

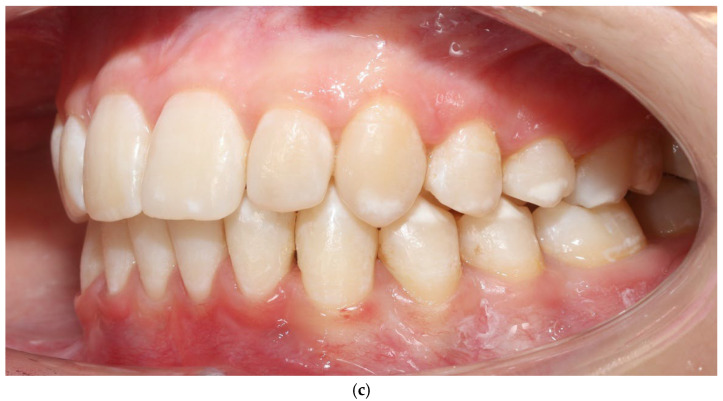
Patient 1 after treatment (**a**–**c**).

The vestibular soft-tissue phenotype appeared thicker, with reduced translucency and a more convex gingival contour. Gingival margin stability was maintained throughout therapy, and no recession, bleeding, or inflammation was present (Figure 4a–c). Outcome assessment in this case report is primarily qualitative and based on standardized clinical photography and clinical phenotype indicators (defined by periodontal probe transparency through the gingival margin), without quantitative soft-tissue thickness measurements.

### 3.2. Patient 2—Soft-Tissue and Intra-Arch Control Outcomes

Because BPS was applied only in the maxillary arch, this patient provided an intra-arch control model under identical biological conditions. The maxillary BPS arch appeared to show features consistent with thickening of the vestibular phenotype, increased soft-tissue resilience, and reduced root translucency. In contrast, the mandibular control arch (treated with conventional biomechanics) appeared to retain a thin, translucent, and flattened phenotype. These qualitative clinical observations from both patients and both arches, including the intra-arch control, are summarized in Table 1.

Gingival phenotype was assessed using a standardized periodontal probe transparency test and evaluation of tissue resistance. No recession, pathological probing depths, or BOP were recorded. Although no ultrasonographic or transgingival thickness measurements were collected, the photographic changes were reproducible and consistent with recognized clinical indicators of phenotype thickening.

A CBCT scan obtained immediately after removal of BPS for qualitative assessment of the buccal cortical outline but was affected by metal-related scatter from brackets and archwire. Because the scan captured only the early remodeling phase, buccal cortical visibility was insufficient for reliable quantification, and no numerical CBCT analysis was performed at this stage. A full post-treatment CBCT will be obtained after orthodontic finishing to allow adequate mineralization of newly formed bone.

**Figure 5:**
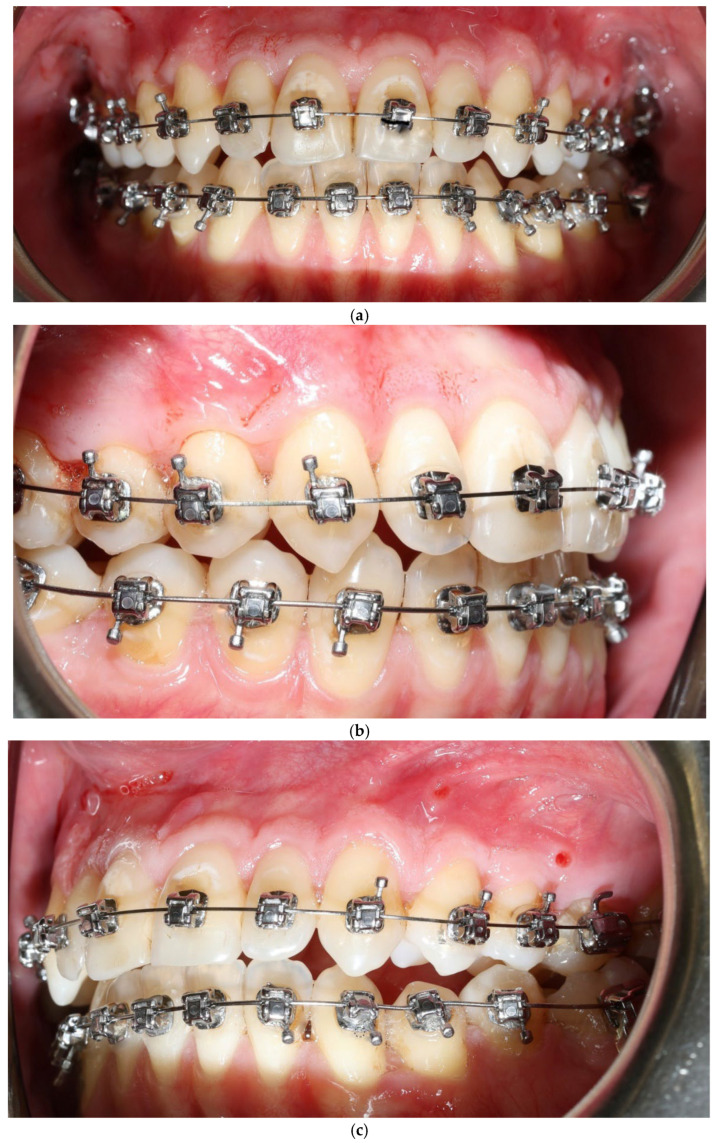
Patient 2 after expansion and BPS removal from the upper arch under treatment (**a**–**c**).

No adverse periodontal events were observed (Figure 5a–c). All changes in soft-tissue phenotype in the BPS arch occurred without any surgical augmentation.

## 4. Discussion

Orthodontic expansion in patients with a thin gingival phenotype and a thin buccal cortical plate poses a substantial biological challenge, with a well-recognized risk of marginal periodontal breakdown. The two cases presented in this report provide consistent clinical observations suggesting that the Bone Protection System (BPS) may be associated with differences in the response of both soft and hard tissues in this high-risk population. The magnitude and consistency of the observed changes, captured through clinical photographs, stand in contrast to the typical patterns described in the literature for similar anatomical conditions. The following discussion is organized according to the mechanobiological, periodontological, and clinical implications of these findings.

### 4.1. Main Clinical Observations and Their Significance

At baseline, both patients exhibited a stable periodontal condition with no pathological pocketing, attachment loss, or bleeding on probing (BOP < 10%). Their elevated periodontal risk stemmed exclusively from anatomy—specifically, the combination of a thin gingival phenotype and narrow alveolar housing—rather than active inflammatory disease. Despite these adverse starting conditions, both patients demonstrated clinically appreciable thickening of the vestibular soft-tissue phenotype and stabilization of the gingival contour during expansion.

In Patient 1, post-treatment CBCT was used qualitatively; the scans appeared to confirm continuity of the buccal cortical outline without signs of fenestration or discontinuity [25,26,38,39]. No quantitative assessment of cortical thickness or alveolar width was performed in this case report.

In Patient 2, serial clinical photographs showed features consistent with progressive thickening of vestibular tissues in the maxillary arch treated with BPS, accompanied by increased convexity and stability of the soft-tissue contour. In contrast, the mandibular arch—treated with conventional biomechanics and serving as an intra-individual control—appeared to exhibit persistent soft-tissue translucency, contour flattening, and no features suggestive of phenotypic reinforcement. This intra-arch discrepancy represents a natural within-subject comparison, in which the biological milieu remained constant between arches while the biomechanical conditions differed. The observed differences may therefore be associated with the presence or absence of BPS-mediated force modulation, rather than reflecting patient-specific biological variability alone.

Together, these observations are consistent with the hypothesis that BPS may be associated with a periodontal response characterized by more adaptive and stabilizing features, rather than a resorptive, compression-dominated pattern, even in patients with high anatomical vulnerability.

Overall, the clinical findings in both presented cases are consistent with the proposed hypothesis that BPS may contribute to improved periodontal safety during orthodontic expansion in thin-biotype patients, potentially by limiting buccal cortical loading and supporting adaptive soft-tissue and bone responses.

### 4.2. Comparison with Available Literature

This case report is intended to examine the clinical and radiographic outcomes of using the Bone Protection System (BPS) during orthodontic expansion in two adult patients with thin gingival biotypes and narrow alveolar ridges. It primarily highlights improved soft tissue thickening and stabilization in BPS-treated arches compared with conventional treatment, suggesting that BPS may enhance periodontal safety without the need for additional augmentation.

Recent studies have explored strategies to modify the periodontal phenotype in orthodontic patients to reduce the risk of gingival recession and support soft and hard tissue adaptation. Kadkhodazadeh et al. (2024) demonstrated that combined bone and soft tissue grafting improved gingival thickness, keratinized tissue width, and vestibular depth, while reducing gingival recession over a 12-month follow-up [40]. Similarly, Tironi et al. (2025) showed that surgically facilitated orthodontics (SFOT), combining bone grafts, platelet-rich fibrin, and collagen membranes, not only accelerated tooth movement but also improved the gingival phenotype and prevented gingival recessions, with stability confirmed at 6-month and 3-year follow-ups [29]. Li et al. (2024) investigated soft tissue augmentation in patients with Class III malocclusion, showing increased gingival thickness, enhanced collagen deposition, and stabilization of the gingival margin during labial tooth movement [41].

While these surgical approaches effectively improve periodontal conditions and reduce the risk of gingival recession, they require more extensive procedures, healing time, and patient compliance. In comparison, the Bone Protection System (BPS) combined with corticotomy offers a less invasive alternative that supports soft and hard tissue adaptation, reduces buccal stress during expansion, and may minimize the need for full surgical augmentation. Its clinical implementation, however, involves several challenges: careful technical planning to optimize force vectors, patient acceptance and education to ensure understanding, engagement, and confidence in the procedure, and thorough clinician training to guarantee precise execution and safe outcomes.

Our observations suggest that BPS-assisted expansion may provide a clinically relevant option for patients with thin periodontal phenotypes, balancing procedural efficacy with moderated invasiveness.

Integrating biomechanical adjuncts such as BPS with selective surgical techniques highlights the potential for personalized treatment planning, optimizing periodontal safety, and minimizing patient burden. Further studies with larger cohorts are warranted to confirm long-term stability and clinical outcomes.

Within this broader context of proposed periodontal phenotype modification strategies, the present cases should be interpreted in light of the well-documented anatomical vulnerability associated with thin gingival phenotypes and narrow buccal alveolar plates.

Patients with a thin gingival phenotype and a narrow buccal alveolar plate are widely recognized as anatomically vulnerable and at increased risk of periodontal complications, underscoring the importance of comprehensive assessment of both soft- and hard-tissue phenotypes [42]. The clinical observations obtained in the present cases diverge substantially from the majority of reports describing orthodontic expansion in patients with a thin gingival phenotype and narrow buccal bone plate. Numerous CBCT and clinical studies have demonstrated that labial tooth movement in a thin alveolar housing is predictably associated with thinning of the buccal cortex, formation of dehiscences and fenestrations, and deterioration of the soft-tissue phenotype [38,39,43]. These adverse outcomes are intensified when tooth movement occurs via uncontrolled tipping, which concentrates compressive stresses along the buccal periodontal ligament [43,44]. As a result, patients with a thin gingival phenotype are statistically more likely to experience marginal tissue damage during expansion, including recession and loss of soft-tissue volume [45,46].

In this context, the radiographic findings in Patient 1 are of particular interest: post-treatment CBCT qualitatively suggested preservation of the buccal cortical plate without signs of thinning or discontinuity. This pattern differs from what has been consistently observed in classical CBCT studies by Garib, Baysal, and others, who documented buccal plate reduction even in mild expansion protocols [19,25,38,39,47]. Similarly, in Patient 2, the intra-individual contrast between the BPS-treated maxilla and the conventionally treated mandible further supports the notion that the phenotype improvements observed here do not represent the natural course of expansion in thin alveolar bone, but rather may reflect differences in the biomechanical environment associated with the use of BPS.

Minor variations in cortical visibility were observed in the mandible, likely reflecting physiological remodeling or early mineralization effects rather than compression-related resorption.

#### 4.2.1. Biomechanics: Tipping vs. Axial Displacement and Periodontal Stress Distribution

Uncontrolled buccal tipping is a primary contributor to buccal bone loss, as it concentrates compressive forces within the cervical buccal PDL and creates localized high-stress zones that initiate osteoclastic resorption [43,48,49,50,51,52,53]. In contrast, a more axial displacement trajectory is generally associated with reduced marginal stress accumulation and a lower risk of dehiscence [53,54,55]. In both cases, the observed phenotype behavior appeared consistent with reduced tipping-dominant mechanics and improved vestibular contour stability.

Corticotomy primarily reduces cortical resistance and may accelerate movement, but it does not inherently redirect force vectors or offload cervical buccal PDL compression [56,57,58,59,60,61]. Mechanobiologically, compression-driven hypoxia promotes osteoclastogenesis and resorption signaling (e.g., RANKL, inflammatory mediators), whereas tension-supported environments favor osteoblastic differentiation and matrix deposition pathways [27,62,63,64,65,66,67,68,69,70,71]. Although our prior FEM simulations (reported separately) offer mechanistic plausibility regarding cervical compression reduction, FEM cannot predict biological outcomes; therefore, the present clinical pattern should be interpreted as hypothesis-generating.

#### 4.2.2. Intra-Patient Control and Phenotype Response (Patient 2)

Patient 2 provided an intra-individual comparison under identical systemic conditions, with BPS applied only in the maxilla and conventional mechanics in the mandible. Clinically, the BPS-treated maxillary arch demonstrated reduced translucency, increased convexity, and improved phenotype stability, whereas the mandibular control arch retained a thin, translucent phenotype with flattened contour. A summary of the qualitative phenotype assessment is presented in Table 1.

Periodontal risk models emphasize that thin phenotype combined with thin buccal cortical plate constitutes a high-risk scenario for marginal breakdown during orthodontic movement [27,28,66]. In this context, the absence of recession or other adverse periodontal events in the BPS-treated segments supports the need for further controlled evaluation of biomechanical modulation strategies in thin-phenotype patients.

### 4.3. Biological and Adaptive Interpretation of Tissue Response

The clinical and radiographic observations made in these cases correspond closely with the well-established biological principles governing orthodontic tooth movement. According to the classical model of tissue response, the periodontal ligament (PDL) and surrounding bone react distinctly to areas of compression and tension. These biological mechanisms allow a refined interpretation of the differences observed between BPS-treated and non-treated segments.

#### 4.3.1. Compression Side vs. Tension Side Dynamics

On the compression side, reduced PDL width produces localized hypoxia, impaired cellular metabolism, and activation of pro-resorptive mediators. Several studies demonstrate that compressive stress upregulates RANKL, TNF-α, IL-1, and prostaglandins, initiating osteoclastic activity and predisposing thin cortical plates to resorption and loss of marginal bone height [62,67,68,69]. This is precisely the mechanism believed to underlie dehiscences and fenestrations frequently observed in patients with a thin buccal plate during uncontrolled expansion.

In contrast, the tension side promotes osteoblastic differentiation, increased production of type I collagen, osteocalcin, and osteopontin, and deposition of new extracellular matrix [62,64,70,71]. These processes facilitate bone formation and improved tissue resilience. The soft-tissue phenotype also responds differently under tension, often demonstrating increased thickness and volume when mechanical conditions are favorable.

The photographic evidence in these cases is consistent with the hypothesis that the use of BPS may be associated with a local mechanical environment that appears more tension-dominant, thereby supporting tissue reinforcement rather than degradation. Segments without BPS influence, especially in the distal mandible of Patient 1, displayed features consistent with compression-driven resorption—highlighting the specificity of the biomechanical effect.

Although our previous FEM simulations offered insight into how BPS may alter the moment–force environment—particularly by reducing cervical buccal compression and promoting a more axial loading pathway—computational modeling cannot predict biological outcomes.

The clinical thickening of the vestibular phenotype observed in these cases may represent a biological response that cannot be anticipated from mechanical analysis alone. FEM described separately provides a mechanistic framework that helps interpret why the tissue response may have differed from what is typically observed in thin-bone expansion, but the present findings remain hypothesis-generating and require future controlled studies for validation.

#### 4.3.2. Why Phenotype Thickening Is a Biologically Exceptional Finding

Thickening of the vestibular phenotype without the use of grafts, soft-tissue augmentation, biomaterials, or regenerative periodontal procedures is a phenomenon rarely documented in the literature. In most reports, expansion in thin alveolar bone is associated with compromised soft-tissue architecture, including increased translucency, contour flattening, and subsequent recession. The natural biological tendency in this anatomical context is toward thinning, not reinforcement.

In the present cases, the opposite occurred: the phenotype became clinically thicker, and the marginal contour more stable. This observation aligns with the hypothesis that BPS redirects forces to create a more axial, controlled root movement pathway, reducing harmful compression while amplifying beneficial tensile strain in tissues predisposed to collapse. Such an outcome is particularly significant because it indicates not only the absence of damage but the presence of a favorable adaptive response. It suggests that the tissue environment, when protected from excessive compression, may possess a greater regenerative capacity than previously recognized in thin-phenotype patients.

These findings from the two documented cases are consistent with a mechanistic rationale that may help to interpret the observed clinical stability and support the interpretation that BPS may play a contributing role in the observed periodontal response, although this interpretation remains hypothesis-generating and requires confirmation in larger, controlled studies.

### 4.4. Distinguishing the Effect of BPS from the Effect of Corticotomy

Corticotomy is a well-recognized adjunctive procedure intended to reduce cortical resistance and accelerate orthodontic tooth movement through activation of the regional acceleratory phenomenon (RAP) [72,73,74,75,76].

Although it facilitates biological turnover, available clinical, radiographic, and histological evidence shows that corticotomy alone does not alter the direction of orthodontic forces, does not reduce cervical buccal PDL stress, and therefore does not protect the buccal cortical plate from compression-related resorption [56,59,61,77,78]. Its role is primarily supportive in terms of movement speed, rather than protective in relation to periodontal biomechanics.

In the corticotomy-only cases without any augmentation clinical examination generally demonstrates no thickening of the vestibular soft-tissue phenotype, no improvement in tissue convexity, and no enhancement of the buccal plate [61]. Despite proper orthodontic mechanics, the phenotype remains thin, and the buccal periodontal tissues show none of the favorable adaptive changes observed in the BPS-treated arches.

This finding supports the mechanobiological conclusion that corticotomy alone is insufficient to induce vestibular phenotype thickening [61] or any thickening of the bone morphotype [26].

These findings are fully consistent with our FEM results, which show that corticotomy—even when performed to cancellous depth—does not significantly offload the buccal portion of the PDL nor redirect force vectors away from the thin buccal plate [56].

In sharp contrast, the arches treated with BPS in the primary cases demonstrated clinically evident thickening of the soft-tissue phenotype and radiographic preservation or even reinforcement of the buccal cortical plate. Because the only procedural difference between the two contexts (BPS-treated vs. corticotomy-only) was the presence of BPS itself, the comparison provides supportive clinical observations consistent with the hypo-thesis that the positive periodontal response observed in BPS-treated arches may be associated with BPS-mediated biomechanical modulation rather than corticotomy alone.

This interpretation is further strengthened by Patient 2, whose maxillary arch (BPS + corticotomy) showed progressive phenotype thickening, while the mandibular arch (corticotomy absent; BPS absent) demonstrated persistent translucency and contour flattening. Both arches existed in the same biological environment, and yet only the BPS-modulated arch displayed a favorable adaptive response. This intra-patient contrast reduces the influence of inter-individual biological variability and supports the interpretation that BPS may play a contributing role in the observed periodontal response, rather than corticotomy alone.

## 5. Clinical Implications

The clinical observations presented in this report suggest that the Bone Protection System (BPS) may be associated with a different loading environment compared with traditional approaches, which may support safer expansion and more controlled displacement, even in patients with thin vestibular bone phenotypes. Based on preliminary observations, BPS may warrant further investigation as a possible adjunct to support safer orthodontic movement in patients with thin periodontal phenotypes. The system was associated with preservation of the buccal cortical plate, despite substantial transverse displacement. This contrasts with outcomes commonly reported for conventional mechanical approaches, where compression-dominant loading frequently results in cortical thinning, fenestration, gingival recession, and unfavorable remodeling in susceptible patients.

A clinically significant finding is the conversion of displacement from tipping-dominant to translation-dominant, a biomechanical behavior that has historically required heavy rectangular archwires or complex auxiliaries. In the cases documented here, translation-dominant movement occurred even with minimal expression of torque, indicating that the governing factor was not the appliance but the global stress redistribution created by BPS. In these cases, BPS appeared to act as a biomechanical element influencing root orientation, even with minimal torque expression. Additional FEM data supporting this mechanism are described in a separate bioengineering manuscript currently under review and cannot be reproduced here for ethical reasons. The preservation of the buccal plate observed clinically conceptually aligns with FEM-predicted patterns of tensile microstrain distribution, which have been proposed in biomechanical models to be associated with angiogenic and osteogenic activity. In this context, these observations may be interpreted as consistent with the absence of gingival recession and the apparent thickening of the periodontal phenotype in areas normally at high risk of resorptive breakdown.

## 6. Limitations

This report, like most case-based publications, is subject to inherent limitations arising from the small number of patients, largely qualitative outcome assessment, and the absence of long-term follow-up. Phenotype assessment was non-blinded and based on clinical inspection and standardized photography, which may introduce observer-related interpretation bias despite efforts to ensure consistency. Observer bias is possible, as phenotype evaluation relied on standardized clinical photographs and clinical inspection. Some authors hold patents related to BPS, which could represent a potential conflict of interest. As the inventors of BPS, we have striven to maintain objectivity by implementing intra-patient controls and carefully adhering to standardized outcome assessments, emphasizing the preliminary and hypothesis-generating nature of these findings, while transparently acknowledging our potential conflict of interest. Readers are therefore advised to interpret the results with caution, recognizing that these observations are limited to the two reported cases and do not allow for generalization. Although the observations in both cases demonstrate clear and consistent trends, they should be interpreted as preliminary, and further studies are needed to validate these findings in larger cohorts. In Patient 2, evaluation of the vestibular phenotype relied primarily on clinical assessment, as the final CBCT examination will only be performed after completion of orthodontic therapy. The absence of full radiographic documentation for this patient limits the ability to quantify buccal cortical changes across both arches.

Another limitation is the lack of three-dimensional volumetric analysis of the soft tissues. Modern tools such as intraoral scanning or CBCT-based soft-tissue segmentation could provide a more precise assessment of volume gains and phenotype stability. Additionally, long-term follow-up beyond the active treatment period is currently unavailable, preventing a definitive assessment of the durability of the observed phenotype thickening and cortical preservation.

Despite these limitations, the presented findings provide a clinical hypothesis and mechanobiological plausibility supporting the potential benefits of BPS for patients with a thin phenotype.

## 7. CBCT Timing and Limitations

In Patient 1 and 2, CBCT evaluation after the BPS phase was limited by incomplete mineralization of newly formed bone and by scatter artifacts due to orthodontic brackets and archwire. Because CBCT primarily captures radiographically mineralized tissues, this early scan was unsuitable for quantitative analysis. It is well-appreciated that early remodeling, including osteoid deposition and woven bone formation, is invisible on CBCT until sufficient mineralization occurs—often several months after biomechanical stimulation. Therefore, only a qualitative confirmation of cortical continuity was obtained. Final quantitative CBCT will be collected after full orthodontic debonding.

## 8. Future Directions

Future research should include a larger cohort of patients to enable statistical evaluation of the effectiveness and predictability of BPS in modulating vestibular tissue response. Completion of post-treatment CBCT for Patient 2 will allow for a comprehensive comparison between arches and further verification of the patterns observed in Patient 1.

Prospective studies incorporating soft-tissue volumetric methods—such as digital intraoral scanning, structured-light surface capture, or CBCT segmentation—would help quantify changes in the soft-tissue phenotype more accurately. Long-term follow-up is also essential to assess whether the observed phenotype thickening and cortical stability persist after treatment.

Ultimately, comparative studies evaluating BPS alongside other adjunctive techniques, such as corticotomy, piezocision, or soft-tissue augmentation, could help define the optimal clinical indications and determine the role of BPS within broader orthodontic and periodontal treatment algorithms for high-risk patients.

### Future Applications in Clear Aligner Biomechanics

Although the present report focuses on fixed appliance therapy, the underlying biomechanical principle of BPS—namely, selective modulation of buccal tipping moments and stabilization of the crown–root trajectory—may have relevance for clear aligner systems. Current aligner protocols often struggle to generate sufficient moment–force ratios to prevent unwanted buccal tipping during transverse correction, particularly in thin-phenotype patients.

In theory, an external or integrated auxiliary capable of selectively augmenting root control during aligner expansion could reduce buccal compression of the periodontal ligament and create a more favorable tension-supported remodeling pattern similar to that observed in our BPS-treated cases. This concept remains hypothetical and requires controlled comparative studies; however, these early clinical observations suggest that the addition of targeted moment stabilization may meaningfully strengthen the biomechanical capabilities of clear aligner therapy in anatomically vulnerable patients.

## 9. Conclusions

The use of the innovative Bone Protection System (BPS) in patients with a thin gingival phenotype may support the preservation—and in some segments, the thickening—of vestibular soft tissues during orthodontic expansion, suggesting a divergent response pattern worthy of further investigation. The intra-individual contrast in Patient 2 confirmed that the favorable periodontal effects appear to result from BPS-mediated modulation of force distribution rather than individual biological variability.

These observations suggest a potential benefit of BPS in enhancing the safety of orthodontic expansion in high-risk patients with thin buccal cortical bone; however, they should be interpreted cautiously. Importantly, further controlled clinical studies are required to confirm these findings, evaluate their long-term stability, and determine the broader applicability of BPS, rather than implying immediate clinical adoption.

## 10. Clinical Relevance

Patients with a thin gingival phenotype and a narrow buccal cortical plate represent one of the highest-risk groups for periodontal complications during orthodontic expansion. Even minimal buccal tooth movement in such anatomically vulnerable individuals may lead to cortical thinning, dehiscence, fenestration, and recession.

The cases presented in this report indicate that the Bone Protection System (BPS) appeared to support the preservation—and in some areas, the thickening—of the vestibular soft-tissue phenotype during expansion, even in the absence of any soft- or hard-tissue augmentation procedures. These findings suggest that targeted biomechanical modulation of force vectors may help maintain periodontal safety and could have a beneficial influence on the biological response during orthodontic movement in patients traditionally considered at elevated risk. BPS may therefore serve as a valuable adjunct to support the predictability and relative safety of treatment in thin-phenotype individuals, while acknowledging that these observations are based on a limited number of cases and are hypothesis-generating.

## Figures and Tables

**Table 1:** Clinical vestibular phenotype qualitative assessment in Patients 1 and 2 based on standardized clinical photographs and clinical inspection.

Parameter	Patient 1—Maxilla (BPS)	Patient 1—Mandible (BPS)	Patient 2—Maxilla (BPS)	Patient 2—Mandible (Control)
Baseline translucency *	High (thin phenotype)	High	High	High
Post-treatment translucency *	Reduced (thicker phenotype)	Reduced (thicker phenotype)	Reduced (improved)	Persistent translucency
**Vestibular contour**	From flat → convex and supported	From flat → convex	From flat → more convex	Flattened contour
Soft-tissue thickness *	Clinically increased	Clinically increased	Clinically increased	No increase
**Gingival margin stability**	Improved	Improved	Improved	Unchanged
**Risk signs (pre-treatment)**	High-risk anatomy	High-risk	High-risk	High-risk
**Risk signs (post-treatment)**	No recession, no thinning	No recession	No recession	Persistent thin phenotype
**Interpretation**	Positive adaptive response (BPS)	Positive adaptive response (BPS)	Marked qualitative phenotype improvement—BPS	Pattern consistent with conventional mechanics in thin phenotype (control).

* Assessment based on visual inspection (using a standardized periodontal probe transparency test) and clinical palpation.

## Data Availability

All data supporting the findings of this study are contained within the manuscript. Additional anonymized patient information is available from the corresponding author upon reasonable request.
